# Eligibility screening older research participants using remote cognitive assessment—experiences and reflections from a primary care randomised controlled trial

**DOI:** 10.1186/s13063-022-06805-9

**Published:** 2022-10-08

**Authors:** Caroline Brundle, Anne Heaven, Andrew Clegg

**Affiliations:** 1 Bradford Institute for Health Research, Bradford Teaching Hospitals NHS Foundation Trust, Bradford, UK; 2 Academic Unit of Ageing and Stroke Research, University of Leeds, Leeds, UK

**Keywords:** Recruitment, Remote, Telephone, Eligibility, Cognitive screening, Older, MoCA, Community

## Abstract

**Background:**

The COVID-19 pandemic forced many research teams to adjust the way they conduct studies, including moving to remote delivery of some or all of their recruitment and data collection processes. The Montreal Cognitive Assessment (MoCA) is widely used in research and is available in multiple formats for different groups and assessment settings. Here, we reflect on our experiences of administering the MoCA Blind/Telephone as part of the initial telephone eligibility check for participation in a randomised controlled trial with community-dwelling older people with frailty.

**Main body:**

In response to COVID-19, a number of changes were made to the trial’s screening and recruitment procedures, to minimise the amount of time the researchers would spend in the participants’ homes when recruitment began in May 2021. One of the changes was for the researchers to conduct a cognitive assessment for eligibility during an initial telephone call, rather than during the subsequent home visit for consent and baseline data collection. We found that in comparison with conducting the assessment in-person, telephone administration caused uncertainty for the researchers about whether participants were struggling to answer questions due to cognition or hearing impairment. Some participants experienced practical difficulties when combining holding a telephone and completing one of the assessment items. It was hard for the researchers to judge the emotional impact that undertaking the assessment was having on the older people on the telephone, without visual warning signs of fatigue or mood. We discuss the potential impact of these issues on trial recruitment and participant engagement, and the feasibility of videoconferencing as an alternative method of conducting the MoCA.

**Conclusion:**

The MoCA is a useful tool when cognitive impairment is part of screening and data collection and it is helpful to have the option to use the test remotely. However, as we have found, telephone testing is not always straightforward. Researchers should weigh up the pros and cons for each individual study, especially those involving older adults. If choosing remote methods, consider the practicality of using videoconferencing and think about the possible impact of telephone assessment on the relationship with the (potential) research participants.

**Trial registration:**

Personalised care planning for older people with frailty ISRCTN16123291 28/08/2020.

## Background

The COVID-19 pandemic has forced many research teams to adjust the way they conduct studies, including moving to remote delivery of some or all of their recruitment and data collection processes. The Montreal Cognitive Assessment (MoCA) [1] is an example of a cognitive screening instrument that is widely used in research as an eligibility screening tool, baseline measure and/or outcome assessment. The MoCA Full test assesses short-term memory; visuospatial abilities; executive functions; attention, concentration and working memory; language and orientation to time and place. It is conducted face to face in-person or using videoconferencing in approximately 10 minutes and is scored out of 30 points. The MoCA Blind/Telephone test is an adapted version for administration by voice only, for use with participants with visual impairment and for remote assessment by telephone. It is similar to MoCA Full but with slight adjustments to the delivery of some items, and other items requiring visual abilities removed, so is scored out of 22 [2].

In the first few months of the pandemic, some of the MoCA’s co-developers highlighted the challenges associated with remote delivery, with particular consideration to hearing loss [3, 4]. Wittich and Philips (2020) [4] note that remote delivery using the telephone introduces some uncertainty to the testing process. For example, the assessor cannot be sure that the participant is not writing down words to remember, using a calculator or checking the date when they are unable to see the participant [4]. It may be impossible to tell if something is “missed” due to perception problems or a lapse of attention on the part of either party [4]. Phillips et al. (2020) [3] argue that participants with reduced hearing will be disadvantaged by the “impoverished conditions” of telephone communication and the reduced range of hearing frequencies. Further difficulties come from the absence of visual cues from the assessor such as smiling and nodding that are important in reassuring people that they are performing well and that they have rapport, and the assessor can also be disadvantaged by the lack of visual cues, for example, signs that the participant cannot hear adequately or are confused or anxious about something [4]. The extra concentration and effort that is required for someone with a hearing impairment when using the telephone may well impact on test performance [3, 4].

Reduced audibility via simulated hearing loss has been shown to significantly affect performance in cognitive assessment, resulting in greater apparent cognitive deficits as audibility decreases [5]. More than 70% of people aged over 70 have hearing loss [6], so the impact of hearing impairment on the use of the telephone for cognitive screening in research studies should not be underestimated. As over 70% of older people with hearing loss are unaware of it [7], assessors will often not be able to factor in the impact of hearing impairment on how they approach testing with many older participants, even if they directly ask about their hearing prior to the start of the test. When the MoCA Blind/Telephone is conducted, those items used in MoCA Full that require drawing or viewing visual stimuli are removed from the assessment. It is acknowledged that deleting vision-dependent items may compromise the test’s validity because certain cognitive domains could be under-represented or unrepresented [8, 9]. A further concern is that the test without visual items could disadvantage participants with hearing impairment, as they may find that items they can use their vision to understand are easier to successfully complete than auditory-only items.

Although the use of videoconferencing with the MoCA Full can avoid some of the difficulties associated with telephone assessment, test administration will be non-standard due to variation in the devices used by the assessor and participant [3, 4]. Camera quality, screen size, internet speed, and lighting may all have an impact. Also, videoconferencing facilities and internet infrastructure are not readily available to all, with lower levels of access for people living in areas of socioeconomic deprivation and rural locations [10, 11].

Studies evaluating the performance of MoCA Blind/Telephone suggest it is an adequate and feasible test of cognition [9, 12], but have been conducted with specific participant groups such as stroke survivors [9] or conclude that further studies with representative populations are needed [12]. Katz et al. (2021) [13] compared performance in telephone and in-person assessments in a community cohort of older people and concluded that the telephone assessment was a valid screen for cognitive impairment. However, the specificity of the MoCA Blind/Telephone conducted on the telephone for identifying mild cognitive impairment (MCI) using the Jak/Bondi actuarial criteria as the reference standard was lower (specificity 0.59) compared with the face-to-face MoCA Full (specificity 0.77), indicating a greater risk of identifying false-positives using the telephone version. The assessors noted any hearing problems they detected whilst conducting the telephone assessments, but the extent of hearing impairment within the cohort is not reported.

Here, we reflect on our personal experiences of administering the MoCA Blind/Telephone as part of the initial telephone eligibility check for participation in the Personalised Care Planning for Older People with Frailty (PROSPER) randomised controlled trial [14].

### Experiences from the PROSPER trial

The PROSPER trial is investigating the impact of a personalised care planning intervention for older people with frailty. In PROSPER the MoCA is used in eligibility screening, with potential participants who score less than 10 on the MoCA ineligible for the trial, but not as an outcome assessment. A proportional cut-off of 7 is used for the MoCA Blind/Telephone assessment.

Following a successful feasibility trial, the main PROSPER trial was due to begin in early 2020, but the COVID-19 pandemic resulted in a significant delay to the start of recruitment. A number of changes were made to the trial’s screening and recruitment procedures, to minimise the amount of time the researchers would have to spend in the participants’ homes when recruitment began in May 2021.

One of the changes made was for the researchers to conduct the MoCA Blind/Telephone test during a telephone call made to older people who had responded to an invitation to participate in the trial whenever possible, rather than the MoCA Full during the subsequent home visit for consent and baseline data collection. Cognitive screening was only conducted once; either on the telephone or during the home visit. In the telephone calls, the cognitive assessment was conducted after discussion about the trial and prior to arrangements being made for the home visit. The PROSPER researchers have extensive experience of using the MoCA Full test face to face with older people, have completed the mandatory MoCA training [15] and are experienced in remote data collection, but conducting MoCA Blind/Telephone was new to all.

### Researcher uncertainty

Researchers could never be completely sure whether a participant was struggling to answer the questions due to cognition or hearing difficulties. Although MoCA instructions stipulate that an item can only be repeated once by the assessors, this was difficult to adhere to when many participants would ask us to repeat something multiple times. These issues are not unique to telephone administration, particularly while we currently wear facemasks during home visits, but hearing problems can be more apparent when face to face and it is sometimes possible to work around difficulties, for example by using hand gestures to clarify certain words. Older people with hearing loss compensate and give meaning to what they are able to hear by watching the speaker’s body movements, gestures, mouth shapes and facial movements [16]. Prior to the pandemic, we certainly found that many participants used lip reading to enhance their hearing when following instructions. Also, when conducting the MoCA at home visits, if certain words were not understood due to hearing impairment or trouble understanding different accents, partners or family members would repeat them and the participant would often then understand. These additional sources of clarification were not possible over the telephone.

### Practical difficulties

One of the adapted MoCA Blind/Telephone items frequently caused practical difficulties. Participants are required to tap their telephone once with a pen to make a sound in response to a certain letter of the alphabet read out by the assessor. Even after an initial practice to check for audibility before starting the test, during the test we sometimes struggled to hear the tapping or distinguish between taps and background noise. Some participants struggled physically to hold the telephone in position and tap. This may be because some modern telephone handsets are relatively small and it can be difficult to consistently tap in the most audible place. Furthermore, it may not be immediately evident where the most audible place is for mobile phone devices, which frequently have a soft protective casing that can prevent an audible sound being made. Also, some older people find it uncomfortable to hold the telephone for long (due to arthritis, pain and other physical conditions), meaning this and/or the unfamiliar action of crossing the arm across the body and trying to hit an unseen target makes it quite a challenging task for some to manage.

### Emotional impact

Compared to face to face assessments, it was hard to judge the emotional impact that undertaking the MoCA was having on the on older people on the telephone because we did not have the visual, nonverbal information. We only knew what the participants chose to tell us. Some comments such as “Oh thank goodness that’s over” were made on completion. One participant reported having “mild Alzheimer’s” during the brief conversation with the researcher prior to the MoCA. They scored just highly enough to be eligible to participate in the trial and so a home visit from the researcher was arranged for a few days later. However, they called the next day to cancel the visit and decline to participate, stating that doing the MoCA had made them realise their cognition was worse than previously thought. The researcher tried to offer reassurance and confirm that they were still eligible for the study if they wished to take part, but they did not want to proceed. Although this scenario is not unique to telephone administration, we believe that if a researcher was present face to face with the participant, they would be better placed to assess how the participant was feeling and try to alleviate concerns at the time.

As the MoCA is used as an eligibility test in this trial, it has to be completed in the early stages of the older people’s contact with the researchers. We found that introducing the MoCA Blind/Telephone test—asking to undertake a cognitive assessment having never met in person—an uncomfortable experience. In our experience rapport and reassurance develop more naturally, even in a short period of time, when face to face. During the testing, we found it tricky to gauge how participants were responding, without visual warning signs of fatigue or mood. Afterwards, we were uncertain if the participant was adequately reassured.

## Discussion

Our experiences with telephone completion of the MoCA as part of an initial eligibility check for a trial involving older people living with frailty indicate that in this context, researchers can experience uncertainty about the source of participants’ difficulties—cognition or hearing impairment—and how to overcome hearing-related difficulties. Older people can struggle with the practicalities of completing some items and both researchers and participants can be uncomfortable with the emotional impact of the testing experience.

Our experiences echoed many of the points made by Phillips et al. [3, 4] about the uncertainty introduced into the testing process by the use of the telephone. Katz et al. [13] reported that performance on the telephone was not affected by difficulties relating to telephone administration that were recorded by assessors (hearing difficulties, suboptimal hearing conditions, diminished attention/motivation, unauthorised use of external sources (e.g. writing down words to remember) and anxiety about performance). However, we believe that this must be somewhat limited by what a telephone assessor is able to perceive and what is disclosed by the participant. When an assessor can see the participant, they are better able to discover difficulties and take steps to overcome them, to minimise the effects of non-cognitive factors on test performance.

The modified tapping item, in which participants must tap their phone with a pen in response to a certain letter of the alphabet being said by the assessor is intended to evaluate attention [1]. However, the physical challenges posed by this task for some older people could negatively influence their performance on this item over and above their attention abilities. This could particularly impact on older people with frailty, as a result of difficulties due to arthritis, Parkinson’s disease and reduced grip strength.

We should take into account the potential emotional impact on participants of hearing impairment and the suboptimal hearing conditions associated with telephone assessment. When the quality of auditory input is reduced, by impaired auditory abilities or by adverse acoustic environments, listeners may expend more mental effort to concentrate, comprehend, remember, and respond. In some situations, when listeners are unable or unwilling to sustain a sufficiently high level of effort, they may experience fatigue and/or decide to quit the task at hand to avoid becoming fatigued [17]. The additional effort taken to concentrate could mean that participants completing the MoCA Blind/Telephone would be more likely to experience greater fatigue and emotional strain than if being assessed face to face, potentially impacting negatively on the person’s attitude towards the research as a whole. A negative experience of completing the MoCA on the telephone might make participants decide not to continue their participation in the study.

We found it difficult to gauge how participants were reacting to being assessed on the telephone. Information on how older people feel after cognitive testing is scarce, but hospitalised older people interviewed after cognitive screening reported finding the screening strenuous, and while some felt pride and relief afterwards, others experienced shame and irritation [18]. Distress has been reported by 47% of cognitively intact older people following cognitive testing, with no predictor of distress identified, and this increased to 70% of participants with Alzheimer’s dementia [19]. This suggests that negative emotional responses are likely to be experienced by older people who complete the MoCA as part of a research study. On the telephone, researchers have a limited ability to identify and respond to the emotional impact of testing compared to when they are face to face with participants.

Many of the issues we experienced are related to the context in which the MoCA Telephone/Blind was used. We believe that the context in which the test is being conducted is critical. During an initial telephone eligibility assessment, there is very little opportunity for researchers to establish any rapport with participants and understand their circumstances. We suspect that our experiences of cognitive testing on the telephone would be different if it was being done as part of a pre-planned investigation, or for follow-up assessment in a study where the researcher, or at least the process, is better understood by a participant. Prior experience of completing the MoCA Full with the researcher present could help many participants feel more at ease with subsequently doing a telephone version and researchers may benefit from understanding the difficulties experienced at previous assessments.

Videoconferencing has been recommended as the preferable method for conducting the MoCA remotely [4]. The MoCA Full test can be completed and video communication alleviates many of the problems associated with telephone testing discussed above. However, videoconferencing requires access to the internet and equipment, user-proficiency and pre-planning - none of which are guaranteed in research with older people and further risking excluding people based on socioeconomic factors. After the first few months of the COVID-19 pandemic, 24% of over-75s in the UK were using the internet more than they did pre-pandemic and 46% of those older people who used the internet were using it to make video or voice calls. However, internet usage mostly increased in those groups who were already using it regularly pre-pandemic and so 42% of all over-75s were not using the internet [20].

While digital engagement may have increased somewhat as the pandemic continued, there will now still be a significant proportion of older people who cannot or do not wish to use the internet. Nearly two-thirds of people without access to the internet in their households say they do not need it or are not interested in it [10]. Thirty-two per cent of people who do not use the internet say there is nothing that could encourage them to get online [21]. Therefore, videoconferencing would only be an option for a subset of older research participants. This is not to say that videoconferencing should be ruled out as a means of delivering the MoCA in all research studies with older people. It could reasonably be offered as an option for baseline or follow-up data collection, when the remote assessments can be scheduled and planned for. Reliance on digital methods alone though could potentially increase inequalities in research participation given the lower levels of digital engagement in areas of socioeconomic deprivation and rural locations [10, 11].

It is encouraging that new iterations of the MoCA for face-to-face use with visual and hearing impairments are being developed [8]. The latter incorporates some adapted items and visual stimuli [22]. The absence of visual stimuli in the telephone MoCA Blind/Telephone may inadvertently disadvantage a participant with hearing impairment and artificially ‘cap’ the number of points they are able to achieve. Indeed, the specificity of the telephone MoCA Blind/Telephone is lower than for face-to-face tests; false positives in the indication of cognitive impairment are more likely with telephone administration [13]. This could mean that people with hearing impairment may be unnecessarily excluded from studies that use cognitive impairment as an eligibility criteria, if assessed via telephone.

## Conclusions

The COVID-19 pandemic has caused many difficulties and delays for community-based research with older people. However, the situation also led to innovation and the exploration of alternative ways of delivering studies with benefits such as: the inclusion of participants who would otherwise be reluctant due to COVID concerns; allowing clinically vulnerable staff to continue working on studies and more efficient logistics when conducting research in rural or widely dispersed communities. Remote methods of communication and data collection will certainly continue to be used in the future. The MoCA is a useful tool when cognitive impairment is part of screening and data collection and it is helpful to have the option to use the test remotely, but as we have found, telephone testing is not always straightforward.

Researchers should weigh up the pros and cons for each individual study, especially those involving older adults. While it may be essential or preferable to continue conducting research using a combination of remote and face-to-face methods, researchers should carefully consider whether it is in the best interests of the individuals involved and the study itself for the MoCA to be used remotely or should it be kept face to face. If choosing to use remote methods, consider the practicality of using videoconferencing and think about the possible impact of telephone assessment on the relationship with the (potential) research participants. Consider the context in which the MoCA Blind/Telephone will be used; it is likely to be most problematic as part of an initial eligibility check and possibly better used as part of follow-up assessments. If it can be undertaken face to face in the first instance, there is more opportunity for rapport to be established, for participants to be generally familiar with processes and for researchers to better gauge and discuss individuals’ ability to complete via telephone at any future assessment time points. We recommend the issues we have highlighted in this paper be further investigated using a study within a trial (SWAT) methodology, assessing the impact of telephone versus face-to-face cognitive assessment for eligibility screening.

**Table:** 

**Considerations for the use of remote MoCA assessment for eligibility:** • What are the pros and cons for the individual study? • Would it be in the best interests of the participants and the study for the MoCA to be used remotely? • Can face-to-face contact be used to develop rapport with participants and to gauge and discuss individuals’ ability to complete via telephone? • What are the practicalities of using videoconferencing with the study population, including access to internet and equipment? • Could people with hearing impairment could be disadvantaged, discouraged or unnecessarily excluded from a study as a result of telephone eligibility assessment? • Could remote testing be used for follow-up assessments, following an initial face-to-face assessment?	

## Data Availability

Not applicable.

## Abbreviations

MOCA: Montreal Cognitive Assessment
PROSPER: Personalised Care Planning for Older People with Frailty
